# Prevalence of and Risk Factors for Community-Based Osteoporosis and Associated Fractures in Beijing: Study Protocol for a Cross-Sectional and Prospective Study

**DOI:** 10.3389/fmed.2020.544697

**Published:** 2020-12-09

**Authors:** Menghua Sun, Yili Zhang, Hao Shen, Kai Sun, Baoyu Qi, Chenchen Yu, Yingjie Zhi, Ranxing Zhang, Junjie Jiang, Yan Chai, Xu Wei, Yanming Xie

**Affiliations:** ^1^Institute of Basic Research in Clinical Medicine, China Academy of Chinese Medical Sciences, Beijing, China; ^2^School of Traditional Chinese Medicine, Beijing University of Chinese Medicine, Beijing, China; ^3^Changxindian Community Health Service Center, Beijing, China; ^4^Wangjing Hospital, China Academy of Chinese Medical Sciences, Beijing, China; ^5^Department of Epidemiology, University of California, Los Angeles, Los Angeles, CA, United States

**Keywords:** osteoporosis, osteoporotic fracture, cross-sectional study, follow-up, Chinese medicine

## Abstract

**Background:** Osteoporosis (OP) patients are usually asymptomatic until osteoporotic fractures occur, which makes early diagnosis and prevention difficult, and the associated fractures secondary to OP could be preventable with appropriate management. Therefore, early identification and relevant evidence-based management of OP could guide the prevention of subsequent fractures. This study will investigate the prevalence of OP and the incidence of osteoporotic fractures in Beijing community residents to further explore the related risk factors and put forward suggestions for people aged 45–80 years old.

**Methods:** Over 2 years, this study will conduct an OP screening and a prospective follow-up in the Beijing community to investigate the incidence of osteoporotic fractures. The study will undertake bone mineral density detection, collect biological samples, and record information via questionnaires.

**Discussion:** The study aims to investigate the potential risk factors for osteoporosis and explore syndromes from traditional Chinese medicine that are associated with this condition based on large samples from the Beijing community. Data on the incidence of osteoporotic fractures among community dwellers in Beijing over the two-years will be available on the Chinese clinical trial registry: ChiCTR-SOC-17013090.

## Introduction

Osteoporosis (OP) is caused by changes in bone microstructure and decreases in bone strength (1, 2). As society has aged, this condition has become a serious issue that affects the physical and mental health of many elderly people (3). The most devastating outcome of OP (4), osteoporotic fractures, also known as fragility fractures, can inflict heavy burdens on families and society more widely, leading to increased use of healthcare resources and economic costs in Asia (5, 6). In China, the annual number of cases of OP-related fractures will be ~5.99 million by 2050, with an estimated cost of $25.43 billion (7).

In recent years, an increasing number of countries have started to be more concerned about community-based public health programs (8–10). Typically, there is a large population with osteopenia or osteoporosis in the community, which are undiagnosed before fractures occur, meaning that community-based health programs for OP screening could be of great significance to residents. For instance, bone mineral density (BMD) and bone turnover marker testing are necessary to ensure preventive interventions to reduce risks for populations with low bone mass, especially postmenopausal women, and elderly men (11, 12). Therefore, community-dwelling middle-aged and elderly people are subjects for whom it is important to prevent and manage OP and its associated fractures.

On the other hand, OP is a chronic disease that has complex causes. Some risk factors have already been confirmed in interviews and community research (13–15). A cross-sectional study in northwestern China found that the prevalence of OP was 9.65% for postmenopausal women and 8.08% for elderly males according to the criteria of the World Health Organization (WHO), and the risk of OP may be associated with age and body mass index (16). However, the epidemiological findings of OP, osteoporotic fracture, and other potential influencing factors were not identical in the different regions (17). A lack of follow-up is common in the majority of community-based studies.

This prospective study will investigate conventional risk factors and relevant symptoms as reported in traditional Chinese medicine (TCM) in populations with a high risk of postmenopausal osteoporosis (18, 19). Based on the findings of this preliminary study, we will then conduct a further cross-sectional survey and a prospective follow-up study in the Beijing community. The present prospective study aims to investigate the prevalence of OP and the incidence of osteoporotic fracture in Beijing community residents to further explore the risk factors related to this condition and to put forward some rational suggestions for patients. This research on “Beijing community-based Osteoporosis and osteoporotic fracture screening: a cross-sectional and prospective study (beyond)” will provide reliable data for the prevention of OP and osteoporotic fractures among community-dwellers in the Beijing area.

## Materials and Methods

**Figure 1:**
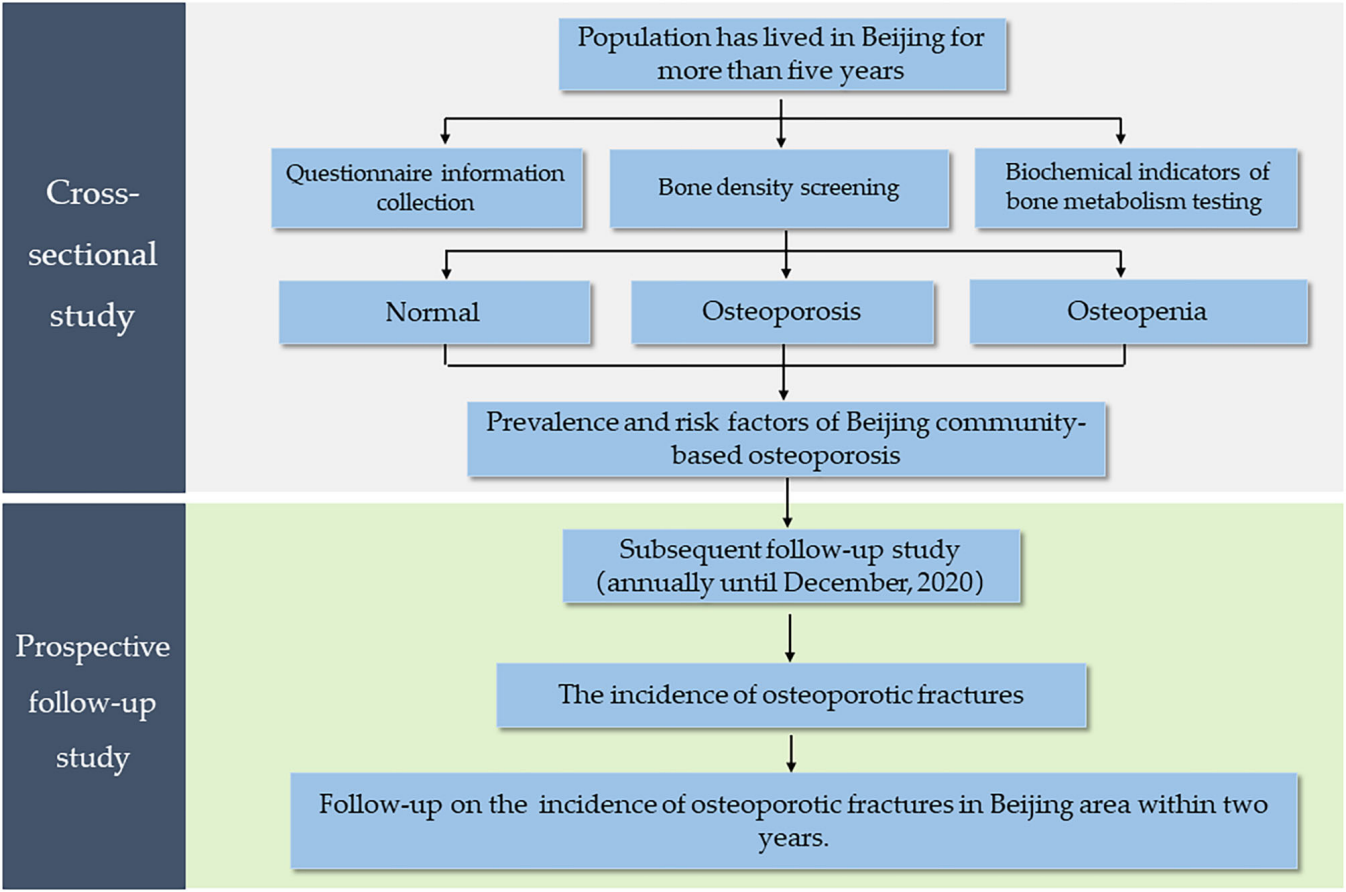
Flow diagram of the study.

This is a community-based cross-sectional and prospective follow-up study. Based on the different economic conditions in Beijing, we will seek residents from several districts from people who respond to an invitation to participate. Then, the participants will be screened, based on predetermined inclusion and exclusion criteria, and an epidemiological investigation will be conducted. The investigation will gather data on BMD screening, biological samples, and questionnaire information. Each participant will be followed for at least 2 years to track incidences of osteoporotic fracture. Based on a power analysis, the required sample size will be at least 1,500 patients. A flow chart of the research process is shown in Figure 1.

### Participant Recruitment

Due to the potential impact of different socioeconomic conditions on the bone mass of populations, this study will recruit participants in the central urban area (Chaoyang District, Beijing) and suburbs (Fengtai District, Beijing). The aim is to avoid the potential bias caused by economic circumstances, education, work, and other factors.

We will randomly select 2 to 5 communities across two administrative regions and then contact the local committee that represents community residents or health service departments. The researchers will work with community staff to post recruitment advertisements in community corridors, billboards, and other public places, which will explain the purpose of this study, testing project, and potential benefits for the participants who take part.

### Ethics and Registration

This study was approved by the medical ethics committee of Wangjing Hospital, China Academy of Chinese Medical Sciences (approval number: WJEC-KT-2017-020-P001) and registered in the Chinese Clinical Trial Registry (approval number: ChiCTR-SOC-17013090). The study will notify all participants or their legal representatives before they are asked to sign an “informed consent form” in accordance with the Helsinki Declaration.

### Diagnostic Criteria

According to the criteria outlined by the WHO and Chinese guidelines for the diagnosis and treatment of primary OP (20), T-score ≥-1 is defined as normal, T-score < −1 and > −2.5 is considered osteopenia, and T-score ≤-2.5 is considered OP based on bone densitometry.

TCM syndrome refers to the nature of a certain stage in the occurrence and evolution of osteoporosis and the specific internal and external environment of the individual/patient at that time. TCM treats a disease based on full consideration of the individual constitution, climatic and seasonal conditions, and environment. This is embodied in the term “giving a treatment on the basis of syndrome differentiation.” Syndrome differentiation is one of the most important factors in the practice of TCM and means diagnosing an illness as a certain syndrome based on analyzing specific symptoms and physical signs, which are collected by inspection of auscultation, olfaction, and palpation. Thus, TCM syndrome differentiation could be used as a method of patient stratification and could help in choosing the most appropriate patients for intervention, based on clinical information on different syndromes.

According to the guidelines on clinical practice for the treatment of primary OP in traditional medicine, the TCM syndromes presented by patients mainly include kidney-Yang deficiency syndrome, liver-yin, and kidney-yin deficiency syndrome, spleen-Yang, and kidney-Yang deficiency syndrome, and blood stasis and qi stagnation syndrome, all of which will be recorded in the present prospective study.

### Inclusion and Exclusion Criteria

The inclusion criteria for this study will be as follows: (1) the individual has lived locally for more than 5 years; (2) female participants will ve aged between 45 and 80 years old (45 ≤ age < 80) and male participants will be aged 50 to 80 years old (50 ≤ age < 80); (3) a written informed consent form will be signed by the subjects, and the process of obtaining this informed consent will be ethical. Participants who suffer from mental illness, such as a mental deficiency, memory or thinking disorder, and who are unable to cooperate with investigators due to disability or lack of physical capacity will be excluded.

### Bone Density Screening

A dual-energy X-ray absorptiometry (DEXA) device (HOLOGIC Wi, United States) will be used to detect the lumbar spine and bilateral hips and to record the *T* and *Z*-values of each site. The precision of the instrument will be set at 1%, that is, the error of repeated measurement will be <1%. After using the instrument every day, professional staff will be responsible for checking accuracy and maintenance.

### Biochemical Indicators of Bone Metabolism Testing

The bone metabolism indicators we will measure will include blood calcium, phosphorus, magnesium, creatinine, alkaline phosphatase, thyroid stimulating hormones, 25-hydroxyvitamin D3, and osteocalcin. Additionally, bone turnover markers, including procollagen type 1 N-terminal propeptide (P1NP) and the β cross-linked C-telopeptide of type 1 collagen (β-CTX), will be determined by electrochemiluminescence.

For each subject, 7 ml peripheral venous blood will be collected in fasting conditions between 8 a.m. and 9 a.m. on the day of physical examination. The anticoagulant vacuum blood collection tube will be 1 ml and the two procoagulant vacuum blood collection tubes will be 3 ml. The Guangzhou KingMed Diagnostics Limited Liability Company is responsible for collecting and testing blood samples.

### Collecting Clinical Information

All participants will complete a face-to-face paper version of the questionnaire and undergo a comprehensive physical examination. The questionnaire and examination will mainly assess the general characteristics of participants, menstrual status, menopausal age, number of pregnancies, number of births, education level, income level, history of smoking and drinking, history of previous illness and medication, number of falls, daily dietary habits, TCM syndrome, and so on. Information on various fractures, such as the number, cause, location, and therapeutic method, will be recorded in detail. The questionnaire will also include the International Physical Activity Questionnaire (21), the 1 Min Osteoporosis Risk Assessment Test of the International Osteoporosis Foundation (22), and the EuroQol 5-dimension instrument (23) to evaluate the physical activity and quality of life of participants.

### Follow-up Study

A subsequent follow-up study will be conducted annually until December 2020.

### Primary Outcomes

This study will be divided into two stages. In the cross-sectional study, our primary outcome was BMD. In the follow-up period, an osteoporotic fracture is the most crucial outcome. Although important information on new fractures will be obtained through the self-reports of participants or interviews during the follow-up, we will immediately re-evaluate and confirm the bone mass of patients with new fracture events based on DEXA. Patients who suffer from fractures will be asked to describe when and why they occurred, their fracture site, and treatment after the fracture.

### Data Management and Statistical Analysis

Data will be recorded in the case report form (CRF), and it will be reviewed and validated by researchers, data managers, and statisticians for final statistical analysis at the end of this study. The CRF will be entered into Epidata 3.1 software by two researchers independently, and the recorded data will be checked for consistency. If there are inconsistencies, the CRF or other original records will be carefully verified. The data will be modified one by one until the independently entered data are identical. The data administrator will further check the exported database and use a logic check to check data until and doubts are resolved. The electronic database will eventually be locked.

IBM SPSS version 22.0 (SPSS, Chicago, IL, United States) software will be used to analyze the data. Continuous data will be analyzed using the independent *t*-test or Wilcoxon's rank sum test, while categorical data will be compared using the chi-square or Fisher's exact test. All statistical analysis tests will be performed using a two-sided hypothesis test. The *P* ≤ 0.05, and the difference in the test will be considered statistically significant. We will also adjust the correlation analyses for factors such as age, gender, and comorbidities.

## Discussion

OP is characterized by low bone mass and microstructured bone tissue degradation (24). The high mortality and morbidity of osteoporotic fractures make them a serious public health threat (25, 26). The incidence and prevalence of fractures also varies across geographic regions and countries (27–30). Therefore, it is important to develop specific and preventive measures. Additionally, the prevalence of OP and associated fractures in the Beijing community has received little attention. The relatively large sample size of the research allows for a systematic investigation of the risk factors associated with OP via the cross-sectional study, including TCM syndrome differentiation and functional activity, which few community studies have previously investigated. Based on the follow-up of fracture outcome, this research will also dynamically monitor the incidence of osteoporotic fractures and explore the influencing factors of osteoporotic fractures in depth through a comprehensive survey.

This study also has several limitations. First, this study may have limited external validity, as it will only include the participants from some districts in the Beijing area. Second, the final results may be influenced by unequal numbers of gender or age groups in the real-world setting, so the clinical practical value will be lower than that of randomized controlled trials. Third, recall bias may arise from some of the retrospective questions, such as patient history relating to previous illness and medication (31). In summary, more useful information on the prevention and control of OP and associated fractures will be beneficial to improving the management of healthcare in the Beijing community.

## Ethics Statement

The studies involving human participants were reviewed and approved by the medical ethics committee of Wangjing hospital, China academy of Chinese medical sciences. The patients/participants provided their written informed consent to participate in this study.

## Author Contributions

XW and YX: protocol design. YC, XW, and YX: design of the statistical plan. YZha and XW: trial registration. MS, YZha, HS, KS, and BQ: data acquisition. YZhi, RZ, and JJ: trial supervision. MS and CY: data cleaning. MS, YZha, and XW: manuscript draft. BEYOND group: manuscript revision. All authors contributed to the article and approved the submitted version.

## Conflict of Interest

The authors declare that the research was conducted in the absence of any commercial or financial relationships that could be construed as a potential conflict of interest.
