# Supplementary material for: Prevalence of and Risk Factors for Community-Based Osteoporosis and Associated Fractures in Beijing: Study Protocol for a Cross-Sectional and Prospective Study
Source: Front Med (Lausanne). 2020 Dec 9;7:544697. doi: 10.3389/fmed.2020.544697 (PMC7757753; doi:10.3389/fmed.2020.544697)
Supplement: Supplementary file 1 [file Table_1.DOCX]

Supplementary Material

Supplemental Table 1 Conditions and diseases that cause or contribute to osteoporosis and fractures

| **Lifestyle factors** | | |
| --- | --- | --- |
| Alcohol abuse | Excessive thinness | Excess vitamin A |
| Frequent falling | High salt intake | Immobilization |
| Inadequate physical activity | Low calcium intake | Smoking (active or passive) |
| Vitamin D insufficiency |  |  |
| **Endocrine disorders** | | |
| Central obesity | Cushing’s syndrome | Diabetes mellitus (types 1 and 2) |
| Hyperparathyroidism | Thyrotoxicosis |  |
| **Gastrointestinal disorders** | | |
| Celiac disease | Gastric bypass | Gastrointestinal surgery |
| Inflammatory bowel disease | Malabsorption | Pancreatic disease |
| Primary biliary cirrhosis |  |  |
| **Neurological and musculoskeletal risk factors** | | |
| Epilepsy | Multiple sclerosis | Muscular dystrophy |
| Parkinson’s disease | Spinal cord injury | Stroke |

Supplemental Table 2 Risk factors for falls

| **Environmental risk factors** | | |
| --- | --- | --- |
| Lack of assistive devices in bathrooms | Obstacles in the walking path | Loose throw rugs |
| Slippery conditions | Low level lighting |  |
| Vitamin D insufficiency |  |  |
| **Medical risk factors** | | |
| Age | Medications causing sedation (narcotic analgesics, anticonvulsants, psychotropics) | Anxiety and agitation |
| Orthostatic hypotension | Arrhythmias | Poor vision |
| Previous falls or fear of falling | Reduced problem solving or mental acuity |  |
| **Neurological and musculoskeletal risk factors** | | |
| Kyphosis | Reduced proprioception | Poor balance |
| Weak muscles/sarcopenia | Impaired transfer and mobility | Deconditioning |
